# Risk Assessment for Children Exposed to Beach Sands Impacted by Oil Spill Chemicals

**DOI:** 10.3390/ijerph13090853

**Published:** 2016-08-27

**Authors:** Jennifer C. Black, Jennifer N. Welday, Brian Buckley, Alesia Ferguson, Patrick L. Gurian, Kristina D. Mena, Ill Yang, Elizabeth McCandlish, Helena M. Solo-Gabriele

**Affiliations:** 1 Department of Civil, Architectural, and Environmental Engineering, University of Miami, Coral Gables, FL 33146, USA; j.black1@miami.edu (J.C.B.); j.welday@umiami.edu (J.N.W.); 2 Environmental and Occupational Health Sciences Institute (EOHSI), Rutgers University, 170 Frelinghuysen Road, Piscataway, NJ 08854, USA; BBuckley@eohsi.rutgers.edu (B.B.); hillyang@eohsi.rutgers.edu (I.Y.); mccandlish@eohsi.rutgers.edu (E.M.); 3 Environmental and Occupational Health, University of Arkansas for Medical Sciences, Little Rock, AR 72205, USA; AFerguson@uams.edu; 4 Civil, Architectural, and Environmental Engineering, Drexel University, Philadelphia, PA 19104, USA; plg28@drexel.edu; 5 School of Public Health, The University of Texas Health Science Center at Houston, El Paso, TX 79902, USA; Kristina.D.Mena@uth.tmc.edu

**Keywords:** cancer, non-cancer, arsenic, oil spill, risk assessment

## Abstract

Due to changes in the drilling industry, oil spills are impacting large expanses of coastlines, thereby increasing the potential for people to come in contact with oil spill chemicals. The objective of this manuscript was to evaluate the health risk to children who potentially contact beach sands impacted by oil spill chemicals from the Deepwater Horizon disaster. To identify chemicals of concern, the U.S. Environmental Protection Agency’s (EPA’s) monitoring data collected during and immediately after the spill were evaluated. This dataset was supplemented with measurements from beach sands and tar balls collected five years after the spill. Of interest is that metals in the sediments were observed at similar levels between the two sampling periods; some differences were observed for metals levels in tar balls. Although PAHs were not observed five years later, there is evidence of weathered-oil oxidative by-products. Comparing chemical concentration data to baseline soil risk levels, three metals (As, Ba, and V) and four PAHs (benzo[a]pyrene, benz[a]anthracene, benzo[b]fluoranthene, and dibenz[a,h]anthracene) were found to exceed guideline levels prompting a risk assessment. For acute or sub-chronic exposures, hazard quotients, computed by estimating average expected contact behavior, showed no adverse potential health effects. For cancer, computations using 95% upper confidence limits for contaminant concentrations showed extremely low increased risk in the 10^−6^ range for oral and dermal exposure from arsenic in sediments and from dermal exposure from benzo[a]pyrene and benz[a]anthracene in weathered oil. Overall, results suggest that health risks are extremely low, given the limitations of available data. Limitations of this study are associated with the lack of toxicological data for dispersants and oil-spill degradation products. We also recommend studies to collect quantitative information about children’s beach play habits, which are necessary to more accurately assess exposure scenarios and health risks.

## 1. Introduction

Oil contains large amounts of mostly organic chemicals, such as alkalines (linear and branched), cycloalkanes, aromatic hydrocarbons (including polycyclic aromatic hydrocarbons (PAHs)), and asphaltenes. Trace levels of metals are also found in oil [1,2,3,4]. When an oil spill occurs, dispersants are commonly used to mitigate spills by lowering the oil’s surface tension causing floating oil to form micelles that are diluted within the water column. These dispersants are a combination of non-ionic surfactant (a fatty acid plus either polyalkoxylated sorbitol or sorbitan) and an anionic surfactant (a sulphonate compound) in a solvent (a glycol, a glycol ether and/or five to ten carbon alcohols) [5]. Immediately after the Deepwater Horizon (DWH) explosion, the primary dispersant applied in mass was known by the trade name Corexit^®^ 9500 followed by Corexit^®^ 9527. Dioctylsulfosuccinate (DOSS) (a sulfonic acid salt) and propylene glycol are found in both Corexit 9500 and 9527. Additional components of Corexit 9500 include sorbitan, butanedioic acid, propanol, and petroleum distillates. Additional components of Corexit 9527 include 2-butoxyethanol [6]. Thus, oil spill chemicals (OSC) consist of a combination of organic compounds, metals, and dispersant chemicals. Due to natural wave patterns, these OSC can wash ashore and mix with the sediments within the intertidal beach zone, which can then lead to potential health impacts for humans participating in recreational activities at affected beach areas [7].

The causes and sizes of oil spills have been changing over the years, with larger spills impacting larger swaths of coastal beaches, thereby increasing the possibility of significant impacts to marine recreational beaches. From the 1970s through to the 1990s, major oil spills in the U.S. were caused by oil tankers or ship collisions. This includes the largest, the Exxon Valdez, where an estimated 40 million liters of oil leaked into Prince William Sound off the coast of Alaska during 1989 [8]. Other major spills include the Argo Merchant*,* which ran aground in 1976, releasing 29 million liters into Buzzards Bay, Massachusetts [9] and the Mega Borg spill in 1990, which released 15 million liters off the coast of Galveston, Texas [10].

Oil spills have more recently been associated with built infrastructure as opposed to moving ships. For example, due to Hurricanes Katrina and Rita in 2005, approximately 30 million liters of hazardous materials spilled from industrial facilities and storage terminals onshore and oil and gas production facilities offshore near New Orleans, Louisiana and Sabine Pass, Texas, respectively [11]. In 2006, an estimated 11 million liters spilled in Calcasieu River, Louisiana from an oil refinery spill [12].

The largest of the most recent U.S. oil spills occurred 20 April 2010 from the DWH oilrig, releasing about 780 million liters. Ultimately, British Petroleum (BP) and its partners—Transocean and Halliburton—were held financially responsible for the spill. The DWH rig is different from earlier traditional oil drilling platforms. The earliest offshore drilling rigs stood in one spot on steel columns as the base. As drilling moved to deeper locations, it became impossible to build rigs capable of supporting themselves from the sea bottom. In response to the newfound difficulties in reaching oil stores, oil companies shifted to new technologies known as “semisubmersible” rigs, where each rig is tethered in place by a set of cables and anchors. However, the spot where DWH was located (known as Mississippi Canyon block 252) was almost 1.5 km deep, requiring new technology by using a set of thrusters to stay aligned [13]. Seven months prior to the spill, a well nearly thirteen km deep was completed through the use of this rig and days before the spill, the well was cemented in its final state. The structure of the rig consisted of two submarine-like hulls plus a deck the size of two football fields above that served as a living and working space for the crew [13]. The DWH oilrig explosion caused the deaths of eleven crewmembers and injuries to an additional 17 individuals. The well was capped 87 days later on 15 July 2010, but not before the incident resulted in the largest oil spill in U.S. history [14].

Although the DWH oil spill originated in the deep ocean about 66 km from the Louisiana coast, the petroleum dispersed and drifted ashore via oceanic and coastal currents and storms that moved the oil-contaminated water and sediments. Approximately 70,000 liters of chemical dispersants were used to disperse the 780 million liters of spilled oil [15]. Recent evaluations also indicate that up to 22% of the total oil spilled was trapped under the water, carried under the water surface as tar, or deposited onto surface sediments [14]. It is estimated that 1728 km [16] to 2113 km [17] of coastline were impacted as a result causing the subsequent closure of 89 beaches [18]. In a study conducted by the Shoreline Cleanup and Assessment Technique (SCAT) teams for the U.S. Coast Guard, the amount of oil on sandy beaches from Louisiana through the western panhandle of Florida was evaluated during January 2011 [19]. They found that Grand Isle Beach in Louisiana was one of the most heavily impacted, with sand samples collected from submerged oil mats containing 17% oil on average, surface residual sand contained 13% oil, and buried sand in the supratidal zone had 8% oil. At highly impacted neighboring beaches tar ball coverage of the beaches was documented at 0.01% to 8.1% [20,21].

The DWH oil spill is unprecedented in the amount of oil released and the length of the shoreline impacted by oil. This observation emphasizes the need to evaluate human health risks from the impacts of OSC along shores during and immediately after spills, as well as over longer time periods. This study evaluates the potential health risk to children following their exposure to OSC in sand at beaches. Although cleanup workers are initially exposed to higher concentrations of oil contaminants, they typically wear personal protective equipment and their exposures are generally monitored. This analysis considers exposures that might occur after initial cleanup efforts focusing on the exposure to children. Children were chosen because they potentially represent a more susceptible population for exposure and vulnerability to adverse health outcomes following a spill [22,23,24]. Their beach play habits put them at higher risk, which include rolling around in the sand, picking it up with their hands, and even ingesting it in some instances [25], increasing the opportunity for exposure. Additionally, a child’s mouth and nose are closer to the ground than that of an adult, increasing their inhalation exposure to pollutants that volatilize or aerosolize from sediments. Toxic exposures can have greater negative impacts as children transition through critical developmental periods, due to their developing organs and systems.

This risk assessment considers both non-cancer and cancer risks and examines oral, dermal and inhalation exposure to different chemicals, focusing on the matrix of sediments, weathered oil, and tar balls at beaches impacted by the DWH oil spill. Sediments are crucial because much of children’s playtime at beaches is within the intertidal zone sands, an area where contaminants tend to accumulate [26,27].

## 2. Methods

This study followed a risk assessment framework [28,29]. This framework included the identification of chemical contaminants (hazard identification), an estimate for the amount of chemical taken in by a hypothetical child based on the given scenario (exposure route assessment), a characterization of the probability of illness given the dose (dose-response), and then a comparison of risk to acceptable levels for each chemical for each type of non-cancer and cancer endpoint (risk characterization). Factors needed for the exposure assessment, dose-response and risk characterization were obtained from the available literature.

### 2.1. Hazard Identification

To identify chemical hazards, this analysis utilized OSC levels measured at beaches during and immediately after the DWH well was capped. We also collected samples from two of the most heavily impacted beaches in Louisiana, five years after the oil spill, to evaluate risks posed by levels observed at this point in time. The process of collection and analysis from these two datasets is explained below. The following demonstrates how risks were hypothesized, tested, and characterized.

To initiate the process of identifying chemicals of concern (CoC), data collected by the U.S. Environmental Protection Agency (EPA) were evaluated. During and immediately after the DWH oil spill in 2010, the EPA measured the concentrations of many chemicals within environmental media throughout Florida, Louisiana, Mississippi and Alabama [30]. The environmental media chosen for evaluation in the current study were those that would accumulate along the shore: sediments and tar, with weathered oil representing an upper limit of OSC concentrations in the sediments. As mentioned above, a study by OSAT [19] found that sediments had significant oil concentrations, up to 17%; thus weathered oil represents an upper bound (by a factor of 5) of what would be expected, on average, under highly oiled conditions at a beach. Since weathered oil is readily visible, its presence can be used to make decisions concerning whether beaches should be opened or closed. The CoCs within sediments, tar, and weathered oil were narrowed down first by excluding any that were measured at below detection limits, then by excluding any chemicals whose maximum concentrations did not exceed the Florida Soil Cleanup Target Levels (SCTLs). The Florida SCTLs are risk-based thresholds for soils in the State of Florida. These thresholds are based on aggregate exposures (children to adult) to soil within residential settings and adult exposure to soil within commercial and industrial settings [31].

Samples were also collected to supplement the existing EPA data. These samples were collected from Grand Isle State Park (GISP) and Grand Isle Beach (GIB) on 23 April 2015 and sent to Rutger’s University for chemical testing. Samples consisted of sand from the intertidal zone, sand from the dune areas, targeted samples from areas that appeared discolored, and whole tar balls. From GISP, five samples were collected; three regular sediment samples (two from the intertidal zone and one from the upper dune), and two targeted samples from areas that had dark stains which appeared to be weathered oil. At GIB, four samples were collected; one from the bottom of the dune, one from the top of the dune, one targeted sample from the bottom of the dune, and a composite tar sample (a mixture of three tar balls that were collected along the bottom of the dune). It was apparent that new sand had been placed in the intertidal zone, so samples at GIB were collected from the bottom and top of the dunes in areas that did not appear to have been covered by new sand.

Sediment and tar samples were analyzed for semi-volatile organic contaminants and metals. The target metals were those that were identified through the EPA dataset as above residential SCTLs. The organic analyses included those identified through the EPA dataset (the four PAHs) plus a general scan of all semi-volatile organic analytes.

Semi-volatile organic contaminants and PAHs in sediment and tar samples were analyzed by gas chromatography ion trap mass spectrometry (GC-ITMS). The gas chromatography system fitted with an electron ionizer (CP-3800, Varian Inc., Walnut Creek, CA, USA) was coupled with an ITMS (Saturn 2200, Varian Inc., Walnut Creek, CA, USA), which was programed to collect data in both full scan and Select Ion Monitoring (for PAHs) modes. The same GC column (DBXLB 40 m, 4 µm, Agilent Technologies, Santa Clara, CA, USA) was used for all organic separations, whether scanning for all unknowns or for specifically quantifying PAHs. Elution of the analytes from the GC column occurred through a temperature program that ranged from at 35 °C to 300 °C over a 40 min chromatographic run. Data was collected using automatic gain control and optimized scan mode. Unknowns were identified via a library search against NIST/EPA/NIH 2012 mass spectral library. PAHs were quantified using a single standard addition spike of a multi-component PAH standard (4S8905 PAH, Supelco, Bellefonte, PA, USA).

Sediment and tar were pre-processed for organics analysis using a solid phase micro-extraction system (Combi PAL system, CTC Analytics AG., Zwingen, Switzerland) to thermally trap analytes on a solid-phase micro extraction (SPME) sorbent needle which houses a fiber containing the solid sorbent (65 μm polydimethylsiloxane/divinylbenzen StableFlex fiber, Supelco, Bellefonte, PA, USA). Analytes were sorbed to the fiber in headspace mode, heating the sample from 55 °C to 75 °C in 0.5 h. The needle was placed into the GC-ITMS injector, which then thermally desorbed the analytes from the fiber.

Metals were quantified by inductively coupled plasma mass spectroscopy (ICP-MS, Nu Instruments AttoM, Wrexham, North Wales, UK) using 300 resolution in deflector jump mode. Sediment samples were pre-processed by placing 5 mL high purity nitric acid plus 2 g of sediment sample (EMD OmniTrace ultra high purity, VWR) into Teflon™ perfluoroalkoxy (PFA) digestion vessels (HP-500, CEM, Matthews, NC, USA). Samples were digested in a microwave (CEM MARS V, Matthews, NC, USA) and programmed to heat in step mode (1200 W 100% duty cycle, heated over 10 min to 165 °C, held for 1 min, heated to 175 °C over 5 min and held for 10 min). The tar composite sample was pre-processed for organics and metals by pooling and homogenization of aliquots from three different tar balls and then digesting homogenized samples (about 0.12 g each) using a 4-step process. Multiple extraction/digestion steps were required to compensate for the large amount of background organic material in the tar sample matrix. In the first 3 steps, samples were heated (1200 W, 100% duty cycle) at a rate of 9 °C/min to 115 °C, 171 °C, and 180 °C. The vessels were cooled and the pressure was released after each step. Safety membranes were replaced after steps 2 and 3. For the 4th step, samples were heated (1200 W, 75% duty cycle) over a period of 20 min to 200 °C and held for 15 min. Upon preparation, the sediment and tar digestates were then introduced to the ICP-MS via nebulization.

All routine quality assurance and quality control (QA/QC) protocols were employed for this work. Specifically, blank QC samples and matrix-matched spikes were employed to ensure quality measurements were obtained. Spike recoveries from sand were used as acceptance criteria for a valid run. Recoveries of between 85%–115% were deemed acceptable. No significant blank values were observed for either the metals or the organics quantitation.

### 2.2. Point-Estimate Computations of Exposure

For exposure assessment, we considered the scenario of a child during beach play within the intertidal zone. There is a scarcity of human exposure factors relevant to this particular scenario, such as hand-to-mouth ingestion rates, the duration of beach visits, and the frequency of beach visits. To address these data gaps, values were either taken from factors established for residential home exposures (e.g., ingestion rates) or based upon best judgment. Additionally, the analysis considered dermal and inhalation exposures to contaminated sand where the analysis focused on the exposure to the solid media within the intertidal zone. Dermal contact and ingestion of contaminated water, although a potential exposure pathway, was not considered here.

Due to the lack of data on human factors, specifically those relevant to beach play scenarios, we limited our computations to point estimates and assumed averages for human exposure parameters, recognizing that there are uncertainties that bound the point estimates and high risks can be potentially missed. For CoC concentration values, we summarized two sets of values, the 95% upper confidence limits (UCL) and the maximum. The 95% UCLs were used for reporting risk. For cases where the UCLs could not be computed due to too few samples or too few samples above detection limits, the maximum values were used. The 95% UCLs were computed using the ProUCL software program (EPA, Washington, DC, USA) using the non-parametric option [32].

## 3. Results

### 3.1. Hazard Identification

Of the 78,773 data points in the EPA database, 14,434 were from in the sediment matrix, 6363 from the weathered oil matrix, and 327 from the tar matrix. Within these various reservoirs, a subset measured at above detection limits for organic compounds and metals. More organic compounds were detected relative to the metals. Although fewer samples were analyzed in a tar matrix, a higher number of organic compounds and metals were detected in this matrix relative to the sediment matrix (Table 1).

For the chemicals that were measured above detection limits, the highest listed concentration for each chemical was identified and utilized to identify a list of CoCs. The maximum values of these CoCs were then compared to the Florida SCTLs. The Florida SCTLs are available for both residential and commercial land uses. Only those that exceeded the Florida SCTLs (Table 2) were included in the analysis. These seven chemicals were benzo[a]pyrene, benz[a]anthracene, benzo[b]fluoranthene, dibenz[a,h]anthracene, arsenic, barium, and vanadium. Among these CoCs, weathered oil showed the highest concentrations for six of the seven. Use of concentrations found in weathered oil thus provides conservative chemical concentration values for the estimation of risk in most cases.

Although the number of data points in the EPA database is very large, the number of samples analyzed for specific constituents is low. This is significant as it impacts the ability to compute the UCLs. For example, no sediment samples were analyzed for arsenic and barium. Over 400 sediment samples were analyzed for vanadium and over 500 samples were analyzed for the organic CoCs. About 50 samples of weathered oil and two to three samples of tar were analyzed. Due to the small number of samples UCLs were not computed for tar samples. For chemicals for which the majority of samples measured at below detection limits (e.g., dibenz[a,h]anthracene) UCLs could not be computed.

Among the chemicals identified in Table 2, arsenic, barium and vanadium are toxic and linked to non-cancer risks. Arsenic has both carcinogenic and non-carcinogenic risks. Arsenic’s non-cancer health effects include a sore throat and skin irritation, such as warts on the palms, soles of the feet, and torso [33]. Acute barium exposure is known to cause gastrointestinal distress and muscular weakness; long-term, chronic exposure to barium causes high blood pressure [34]. Acute exposure to vanadium causes irritation of the respiratory and gastrointestinal tracts, while exposure over long periods of time can cause degeneration, squamous metaplasia, and benign changes in epithelial tissue [35].

Cancer risks are associated with arsenic, benzo[a]pyrene, benz[a]anthracene, benzo[b]fluoranthene, and dibenz[a,h]anthracene. Arsenic causes skin, lung, and bladder cancer [36]. Benzo[a]pyrene has been linked to lung cancer [37,38] while benz[a]anthracene has been linked to skin and liver cancer [39]. Benzo[b]fluoranthene is expected to be a carcinogen in humans as it is known to cause skin, lung, and liver cancer in animals [40]. Similarly, dibenz[a,h]anthracene causes oral and throat cancer in animals, and is therefore believed to be carcinogenic in humans [41].

The samples taken from GISP and GIB were tested for the chemicals found to exceed the residential SCTLs in the EPA dataset. These included the analysis of metals (arsenic, barium, and vanadium) and PAHs (benzo[a]pyrene, benz[a]anthracene, benzo[b]fluoranthene, and dibenz[a,h]anthracene). In addition to the PAHs, general scans of semi-volatile organic compounds were also run on these samples.

**Figure 1:**
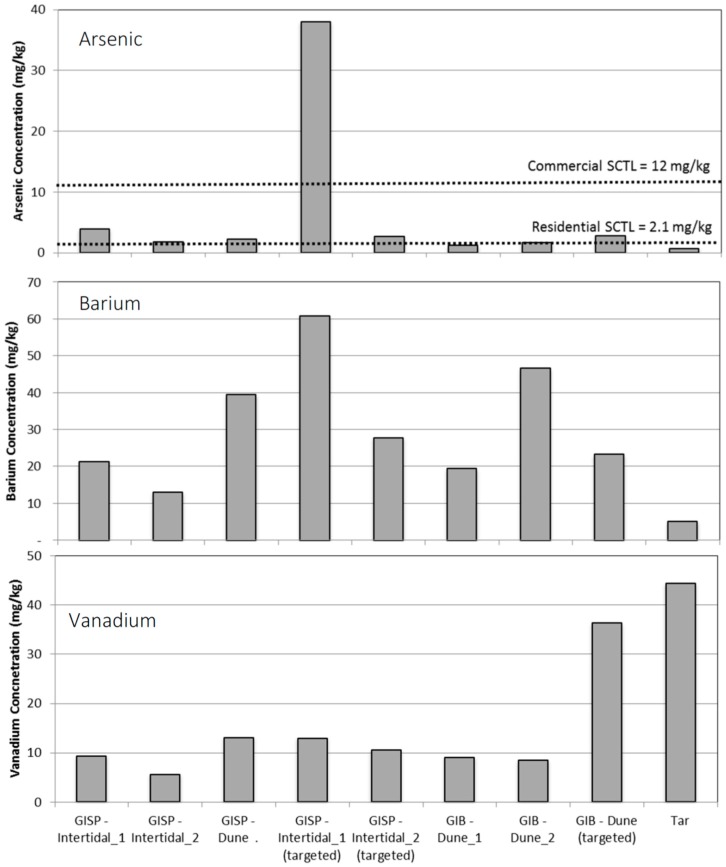
Arsenic, barium, and vanadium levels (mg/kg) in samples collected from Gulf Island State Park (GISP) and from Gulf Island Beach (GIB).

Metals results for samples collected from GISP and GIB show that among the three metals, only arsenic exceeded the SCTL (Figure 1). Five of the eight sediment samples exceeded the residential arsenic SCTL. The sample showing the highest arsenic value was GISP-Intertidal (targeted), meaning it was found in visibly stained sand in the Grand Isle State Park intertidal zone. This sample was above the commercial SCTL and also contained the highest level of barium, although the barium measured below the SCTLs. Of interest was the metal composition of the tar sample showing elevated levels of vanadium, although below the residential SCTL. The targeted dune sample from GIB also had higher vanadium levels relative to other sediment samples.

Differences are observed when comparing the metals results from the GISP and GIB samples to those documented in the EPA dataset (Table 2). The GISP and GIB samples showed detectable levels of arsenic and barium in the sediments, although these metals were not measured as part of the EPA dataset. The tar from GIB showed higher levels of vanadium relative to the two vanadium data points for tar samples included in the EPA dataset. These results are of interest, given the long period of five years that passed since the oil spill, suggesting either naturally elevated background metals levels for these beaches or long-term persistence of these metals. Although the EPA dataset is very expansive, few sediment and tar samples were analyzed for metals, and this may help to explain the higher levels of some metals observed in the samples collected from GISP and GIB through the current study.

Organic analysis, focused on the target PAHs, showed that samples collected at GISP and GIB beaches were below detection limits (<0.0002 ng/g for benzo[a]pyrene, <0.0058 ng/g for benz[a]anthracene, <0.0010 ng/g for benzo[b]fluoranthene, <0.014 dibenz[a,h]anthracene) for the four target PAHs. In addition to the target CoCs, organic by-products were detected at nanogram per gram levels in the GISP and GIB samples (Supplemental Table S1). Many compounds appeared to be oxidative decomposition by-products of OSCs. While the PAHs targeted were at or below the detection limits for those analytes, the number of low level compounds identified with this method (See Supplemental Table S1) demonstrates a possible legacy of previous contamination from the Deep Water Horizon spill. Although none of the PAHs found in the samples collected from beaches could be reliably identified, the large number of hydrocarbons (e.g., decanes and substituted decanes), aromatics (benzenes and substituted benzenes), and oxidized hydrocarbons (alcohols, ketones, etc.) suggest multiple breakdown products associated with a petroleum product. It is possible that this represents the size and volatility fraction most readily retained on sand as a sorbent. As this profile of organic contaminants changes with time, so does the risk associated with exposure. It is expected, however, that the volatility of the compound profile diminished with time and that most of the compounds detected have already reached equilibrium or a state of very slow oxidation/removal with their environment. Overall, the estimated concentrations for these compounds were very low but the results also demonstrate the persistence of the by-products in the beach play environment.

### 3.2. Exposure Assessment

Through standardized equations [28], risks posed by each individual chemical were assessed. Specifically, the algorithms used for computing exposure doses via oral, *D_s_*, dermal, *D_d_*, and inhalation, *D_i_*, routes are given by Equations (1)–(3) below, respectively.

(1) $$D_{s}=\frac{C_{s}\;\times IR_{s}\;\times RBA\;\times \;EF\;\times \;CF}{BW}$$

(2) $$D_{d}=\frac{C_{s}\;\times SA\;\times AF\;\times ABS\;\times \;EF\;\times \;CF\;}{BW}$$

(3) $$D_{i}=\frac{C_{s}\times \left(\frac{1}{PEF}\right)\;\times IR_{a}\;\times ET\;\times EF}{BW}$$

Some exposure factors were constant regardless of the exposure route (oral, dermal, inhalation) or chemical considered. For example, body weight (*BW*), shown in Table 3, corresponded to average weights of children 2 to 10 years of age [42]. Exposure duration (*ED*), averaging time (*AT*), and frequency of exposure (*F*) were used to compute the exposure factor (*EF*) (Equation (4)). Very limited data were available for these factors. For purposes of this study, an *F* value of 12 per year was used. This number is based on the assumption that people will go to the beach twice a month during the warmer months of the year. For non-cancer risks, *ED* was estimated over a period of 1 year and *AT* was also set to 1 year such that *EF* = *F*. For cancer risks, *ED* was estimated over a period of 8 years, going along with the age range of 2 to 10 years, considered to be the most vulnerable child population and likely to be the most active in sand-play. *AT* was assumed over an average lifetime of 78 years [42] (Table 3). The value of 78 years was converted to days in order for the units to cancel.

(4) $$EF=\frac{F\;\times ED\;}{AT}$$

Factors specific to the exposure pathway included soil intake rate (*IR_s_*) for oral routes. The soil intake rate utilized for this study corresponded to a child with an eating condition known as pica, where the child persistently ingests nonfood items [43]. Pica is common in children between the ages of 2 and 3 [44]. Skin surface area (*SA*) and the adherence factor (*AD*), factors specific for dermal routes correspond to children from ages 2 to 10. Skin surface area was calculated from the values given for trunk, arms, hands, legs and feet for the 95th percentile values. The only value excluded from this calculation is the surface area of a child’s head. For computations evaluating dermal risks from tar balls, only the hands and feet were considered. Adherence factors were available in the US EPA Exposure Factors Handbook [42] for mud and sediment based upon specific areas of the body. The values varied from 0.49 mg/cm^2^ for sediment to 47 mg/cm^2^ for mud adherence on hands. For the legs, the values varied from 0.70 for sediment to 23 for mud adherence, respectively [25,45]. Sand adherence during beach play is likely somewhere between the values for sediment and mud, and for this reason, an average value of 18 mg/cm^2^ was used. For inhalation routes, the soil-to-air particulate emission factor, *PEF*, corresponds to the amount of sediment in the air and the value utilized is the default value, which is based upon an assumption of wind-generated suspended sediment particles [31]. The inhalation rate, *IR_a_*, corresponds to children ages 2 to 10 [42]. The exposure time (*ET*) is based upon an assumption for the hours per beach trip (Table 3).

Additional factors that were dependent upon the chemical under consideration included the relative bioavailability factor (RBA) for oral exposure routes, absorption factor (ABS) for dermal routes, and slope factors for all routes (Table 4). These values [31] include a relative bioavailability for arsenic of 0.33 (fraction absorbed), which is based upon a non-human primate model [46]. This value is consistent with the 2010 EPA study [47], which found RBA values from 10% to 60%.

### 3.3. Dose-Response

The computed doses (Table 5) were then used to evaluate non-cancer and cancer risks. For non-cancer risk, the hazard quotient was computed; this is the ratio between the computed dose and the minimum risk levels (MRLs, Table 6) [48]. A hazard quotient greater than 1 indicates a potential chronic non-cancer risk. MRLs were available for only a subset of the exposure routes for arsenic, barium, and vanadium. Among all of the metals evaluated for non-cancer risks, none exceeded a hazard quotient of one. All hazard quotients were less than 0.03.

For cancer risks, once the exposure dose for each chemical and pathway was calculated, it was then multiplied by the cancer slope factor specific to the chemical for each of the three routes (Table 4). Relative toxicity factors were used for the PAH compounds (benzo[a]pyrene 1.0, benz[a]anthracene 0.1, benzo[b]fluoranthene 0.1, dibenz[a,h]anthracine 0.01 [31]). This computation resulted in the final risk values (Table 7).

### 3.4. Risk Characterization

Computations show all hazard quotients below one. Arsenic, barium, and vanadium were not found in high enough concentrations to exceed the MRLs; no adverse effects are anticipated due to acute or subchronic exposures. Increased cancer risks were variable, but considered collectively, represent a very low increased risk from an impacted beach. Increased risks on the order of 10^−6^ are considered extremely low increased risk [49]. No chemicals were on the order of 10^−5^ Arsenic was found to be at extremely low risk levels in sediments for oral and dermal routes of exposure. The PAHs benzo[a]pyrene and benz[a]anthracene were at extremely low increased risk (10^−6^) for weathered oil via dermal routes. For tar, no chemicals were observed at excess risk levels above 10^−6^.

## 4. Discussion

The results from this study suggest that weathered oil measured immediately after the spill has at least seven CoCs that exceed risk-based regulatory guideline levels. These chemicals are the metals arsenic, barium, and vanadium, and the PAHs benzo[a]pyrene, benz[a]anthracene, benzo[b]fluoranthene, and dibenz[a,h]anthracene. Comparison of the EPA dataset against those collected through the current study (five years after the spill) found metals at similar levels, with the exception of vanadium in tar balls. PAHs were below detection limits with evidence showing potential oxidative by-products of organic oil-related compounds within the beach environment. Our risk assessment resulted in hazard quotients less than one, suggesting no adverse effects due to acute or subchronic exposure to weathered oil, impacted sediments, or tar balls. Computations for weathered oil also show extremely low excess cancer risks for arsenic, benzo[a]pyrene, benz[a]anthracene through dermal routes of exposure, and for arsenic through oral routes. Overall, results suggest that health risks are extremely low, even for weathered oil, given the limitations of the available data.

Though the results appear to reflect extremely low risks with respect to known CoC chemicals, there are many unknowns in this study. Analyses of sand samples collected from Grand Isle State Park and Grand Isle Beach suggest a considerable amount of oxidative by-products from the oil. The presence of oxidative by-products has been documented by others with conversion facilitated by biodegradation and photooxidation [50]. The majority of these by-products are of unknown toxicity and so more research is needed to document the potential health impacts of degraded weathered oil chemicals. Although the number of tar ball samples analyzed for metals was low, of interest is that the level of vanadium in the tar ball composite sample collected five years after the spill was high in comparison to the EPA samples. This difference is likely due to the heterogeneity among tar balls composition as the barium levels remained consistent in the tar ball samples between the two datasets. Heterogeneity among tar balls has been well documented at neighboring highly impacted beaches with degradation of the organic components a function of their location on the landscape [21,51]. Landscape location was concluded to control biogeochemical factors such as moisture, salinity, nutrients, which impact the process of microbial degradation [20]. Additional limitations were associated with the lack of information concerning dispersants. Studies suggest the persistence [52] and aquatic toxicity [53] of dispersants to deep-water coral [54], marine zooplankton [55,56], oysters [57,58] and fish [59]. Chemicals trapped in tarmats inhibited cell viability in the hippocampal, kidney and epithelial cells, suggesting human toxicity [60]. Although evidence exists to suspect toxicity, dispersants were not included in our analysis because of a lack of relevant human toxicological data. No dose- response curves were available to assess the human health impacts resulting from the dispersants. Additionally, SCTLs are not available for dispersant chemicals with the exception of propylene glycol, which is characterized by high SCTLs, on the order of 100,000 mg/kg. Therefore, the majority of the dispersant chemicals could not be included in this risk assessment. Actually, the SCTLs need to be expanded to evaluate more chemicals, as some of the chemicals in the EPA sample database were not listed. Of particular interest would be to develop toxicological parameters for 2-butoxyethanol, one of the ingredients of Corexit 9527 which has been linked to liver cancer and reproductive disorders [61]. Of interest would be to evaluate the distribution of illnesses throughout the Gulf Coast areas impacted by oil spill contaminants through standard epidemiologic approaches.

Another prominent issue with determining the risks associated with each chemical was the inability to quantify ranges of human activity factors, specifically the behaviors of children at beaches. Information obtained from residential soil exposure scenarios was used to estimate beach sand exposure. It is emphasized, however, that children’s play behaviors are distinctly different between residential soils versus beach sands and certain micro-activities (i.e., contact rate with soil, mouthing activities) may differ leading to potentially higher exposure and health risks [62,63,64]. At the beach, children are expected to play in the sand and toys are made specifically for this purpose, whereas in residential settings the children’s contact with soil may not be as intimate as at the beach. Thus, there can be considerable error in extrapolating ingestion, dermal contact, and inhalation exposures from residential scenarios. In order to increase the certainty of exposure estimates, a micro-activity study of children’s play activities is recommended. Micro-activity studies use video-recording methods and then convert this video-recorded information into quantitative data on hand-to-mouth behavior and the amount of skin contact during water versus sand-play activities. Additionally, there is no information on how frequently the average family goes to beaches or the duration of beach visits (i.e., macro-activity data). So in addition to micro-activity studies, a macro-activity survey is recommended to evaluate time spent at the beach. The exposure duration for this experiment was assumed to be eight years, based on the period children will be exposed to the beaches while chemicals are present. The dearth of information concerning child play behavior and the frequency and duration of exposure is contrasted by the incredible level of effort placed into defining the environmental chemical concentrations (over 78,000 data points collected from environmental media). Play behavior is also particularly important to document due to the variable distribution of oil at impacted beaches. Studies have shown that submerged oil can be significant and that the vertical distribution of oil in the intertidal zone is also variable and dependent upon hydrodynamic conditions [65]. A study at a neighboring beach found layers of oil in the intertidal zone extending from depths of 15 to 42 cm [66]. Given that children’s play activities at beaches include digging, at times to considerable depths, the vertical distribution of oil should be coupled with knowledge of the children play activities to evaluate potential exposures to buried oil. Overall, additional detailed information would allow the consideration of variability, uncertainty, and probability distributions for population activity factors. Due to the lack of data, the uncertainty associated with these factors is large and believed to drive the uncertainty in the risk estimates computed through the current study. Thus, this study is limited due to point estimates and assumed averages due to the lack of data for children’s play activities within beach settings.

Given the many unknowns associated with this study, we recommend a conservative approach, where recreational beach users avoid beaches with visible signs of weathered oil. Lamendella et al. [14] visited affected beaches two months after the spill and described the appearance of a layer of viscous oil completely covering the sampling site, including large, amorphous globules of oil washing onto shore. Two weeks after that, the beach no longer contained visible globules of oil, but rather small, dried ones. One month after the initial visit and three months after the spill itself, oil and oil mixed with foam were evident and the beach surface presented a light sheen of oil on the surface [14]. What our risk assessment suggests is that beachgoers avoid areas where they can see visible globules of weathered oil because there are still health risks, albeit the available toxicological data suggests these risks are low.

## 5. Conclusions

Overall, the risk assessment conducted in this study should be considered preliminary and specific to the nature of the DWH oil spill. The data utilized corresponded to a very large spill that occurred more than 66 km offshore allowing for considerable weathering of the oil prior to beaching. The long weathering period and the type of oil could play a role in the low risks computed in this study, and may not be representative of ship and tanker spills, such as the Exxon Valdez, the Argo Merchant, or the Mega Borg, which occurred closer to shore, nor would it be representative of the failure of onshore structures, such as those associated with spills caused by Hurricanes Katrina and Rita, and the oil refinery spill in the Calcasieu River. The weathering characteristics from the DWH event were unique. The nature of the release and the distance of the spill from shore resulted in considerable changes in the OSC properties prior to making landfall, allowing for the loss of many of the lighter aromatic components and permitting some degradation of the more persistent components [67]. As a result, the OSCs from this spill were below 10^−5^ or very low excess risk levels for the compounds for which toxicological data were available.

The DWH oil spill had negative impacts on the Gulf Coast area, through its ecological effects and resulting economic losses. Due to the unknowns surrounding risk in visiting contaminated beaches and swimming in contaminated waters, many residents and tourists decided to avoid Gulf beaches altogether, whether they were impacted or not. Improved risk assessments will provide a basis for clearer messages for the Gulf community. This will make them capable of making better-informed choices during and immediately after an oil spill occurs. For this reason, we recommend research be conducted that focuses on quantifying the many parameters that must be used when computing risk from oil spills. Research is particularly needed on adding to the known toxicological properties of oil spill chemicals and on human activity factors specific to beach-related activities.

## Figures and Tables

**Table 1:** Number of chemical measures in sediments, weathered oil, and tar collected by the EPA during and immediately after the DWH oil spill [30].

Category of Chemical Measure	Organic	Metals	Total
Sediment, Total No. of Measurements = 14,434			
Number of Chemicals	33	2	35
Number of Chemicals Above Detection	27	2	29
Weathered Oil, Total No. of Measurements = 6363			
Number of Chemicals	137	25	162
Number of Chemicals Above Detection	52	21	73
Tar, Total No. of Measurements = 327			
Number of Chemicals	152	20	172
Number of Chemicals Above Detection	30	14	44

**Table 2:** CoCs or chemicals found within the EPA dataset at or above Florida’s residential SCTLs. The maximum values and 95% upper confidence limits (UCLs) from the EPA dataset and from the GISP and GIB samples collected 5 years later are shown. The values in bold correspond to levels that exceed Florida residential SCTLs.

Chemical of Concern	Florida SCTL (mg/kg)		EPA Dataset (mg/kg)							GISP and GIB Samples (mg/kg)			
	Residential	Commercial	Sediments			Weathered Oil			Tar ^d^	Sediments			Tar ^d^
			Max	UCL	N	Max	UCL	N	Max	Max	UCL	N	Max
Arsenic	2.1	12	NM ^a^	NA ^b^	0	**39.4**	4.37	51	BDL ^c^	**38.0**	**28.1**	8	0.7
Barium	120	130,000	NM	NA	0	**164**	26.9	49	5.8	60.9	56.4	8	5.1
Vanadium	67	10,000	**77**	27.1	427	25.5	4.6	49	0.2	36.4	21.6	8	44.4
Benzo[a]pyrene	0.1	0.7	**1.49**	0.0255	588	**7.19**	0.808	55	BDL	BDL	NA	8	BDL
Benz[a]anthracene	1.3	6.6	**1.91**	0.0348	588	**33.9**	4.11	53	BDL	BDL	NA	8	BDL
Benzo[b]fluoranthene	1.3	6.5	**1.46**	0.0245	588	**4.4**	0.503	53	0.62	BDL	NA	8	BDL
Dibenz[a,h]anthracene	0.1	0.7	**0.11**	0.00249	588	**0.13**	NC^e^	53	BDL	BDL	NA	8	BDL

^a^ NM = Not Measured; ^b^ NA = Not Applicable; ^c^ BDL = Below Detection Limits. BDL values were variable within each chemical category; ^d^ UCL were not computed for tar samples because the numbers were too low. Only 2 tar samples were analyzed for metals and only 3 tar samples were analyzed for the organics in the EPA dataset. Only one tar sample was analyzed through the current study; ^e^ NC = Not Computed because only one sample was above detection limits.

**Table 3:** Factors utilized for exposure assessment that are independent of chemicals considered.

Factor	Amount	Source
All Pathways		
Body Weight, *BW* (kg)	25.4	[42]
Frequency of Exposure, *F* (days/year)	12	Assumption
Exposure Duration, *ED* (years)	8	Assumption
Averaging Time, *AT* (days)	365 (for non-cancer) 28,489 (for cancer)	[42]
Exposure Factor, *EF* (unitless)	0.263 (for non-cancer) 0.002948 (for cancer)	Calculation
Oral		
Soil Intake Rate, *IR_s_*(mg/day)	1000	[42]
Conversion Factor, *CF* (mg /kg)	0.000001	Calculation
Dermal		
Skin Surface Area, *SA* (cm^2^/event)	11,350	[42]
Adherence Factor, *AD* (mg/cm^2^)	18	[31]
Conversion Factor. *CD* (mg/kg)	0.000001	Calculation
Inhalation		
Soil-to-Air Particulate Emission Factor, *PEF* (m^3^/kg)	1,240,000,000	[31]
Inhalation Rate, *IR_a_* (m^3^/day)	9.62	[42]
Exposure Time, *ET* (hours/day)	3	Assumption

**Table 4:** Chemical dependent factors for oral, dermal and inhalation exposure routes. All factors in this table came from CEHT [31], which provide references to primary sources.

Factor	Oral		Dermal		Inhalation
	Relative Bioavailability Factor, *RBA* (Fraction)	Oral Slope Factor (kg·day/mg)	Absorption Factor, *ABS* (Unitless)	Dermal Slope Factor (kg·day/mg)	Inhalation Slope Factor (kg·day/mg)
Arsenic	0.33	1.5	0.001	1.579	15.05
Barium	1.0	NA ^a^	0.001	NA	NA
Vanadium	1.0	NA	0.001	NA	NA
Benzo[a]pyrene	0.5	7.3	0.01	14.6	3.1
Benz[a]anthracene	0.5	0.73	0.01	1.46	0.31
Benzo[b]fluoranthene	0.5	0.73	0.01	1.46	0.31
Dibenz[a,h]anthracene	0.5	7.3	0.01	14.6	3.1

^a^ NA = Not Applicable.

**Table 5:** Dose in units of milligrams of chemical per kilogram body weight per day computed for chemicals of concern for daily (non-cancer endpoint) and lifetime (cancer endpoint) exposures. Routes evaluated included oral, dermal and inhalation. Concentrations used include the maximum value and the 95% UCL.

Dose (mg/kg/day)	Oral, *D_s_*				Dermal, *D_d_*		Inhalation, *D_i_*	
Exposure Averaging Time	Daily		Lifetime		Lifetime		Lifetime	
	Max	UCL	Max	UCL	Max	UCL	Max	UCL
Arsenic	1.4 × 10^−4^	9.6 × 10^−5^	1.5 × 10^−6^	1.1 × 10^−6^	9.3 × 10^−7^	6.7 × 10^−7^	3.6 × 10^−11^	2.5 × 10^−11^
Barium	1.7 × 10^−3^	5.8 × 10^−4^	1.9 × 10^−5^	6.5 × 10^−6^	3.9 × 10^−6^	1.3 × 10^−6^	1.5 × 10^−10^	5.1 × 10^−11^
Vanadium *	8.0 × 10^−4^	2.8 × 10^−4^	8.9 × 10^−6^	3.1 × 10^−6^	5.8 × 10^−7^	6.4 × 10^−7^	6.9 × 10^−11^	2.4 × 10^−11^
Benzo[a]pyrene	3.7 × 10^−5^	4.2 × 10^−6^	4.2 × 10^−7^	4.7 × 10^−8^	1.7 × 10^−6^	1.9 × 10^−7^	6.5 × 10^−12^	7.3 × 10^−13^
Benz[a]anthracene	1.8 × 10^−4^	2.1 × 10^−5^	2.0 × 10^−6^	2.4 × 10^−7^	8.0 × 10^−6^	9.7 × 10^−7^	3.1 × 10^−11^	3.7 × 10^−12^
Benzo[b]fluoranthene	2.3 × 10^−5^	2.6 × 10^−6^	2.6 × 10^−7^	2.9 × 10^−8^	1.0 × 10^−6^	1.2 × 10^−7^	4.0 × 10^−12^	4.5 × 10^−13^
Dibenz[a,h]anthracene	6.9 × 10^−7^	1.3 × 10^−8^	7.7 × 10^−9^	1.4 × 10^−10^	3.2 × 10^−8^	5.9 × 10^−10^	1.2 × 10^−13^	2.2 × 10^−15^

* Daily dose for vanadium through inhalation routes is 6.9 × 10^−11^ mg/kg/day using the maximum value and 1.9 × 10^−11^ mg/kg/day using the 95% UCL.

**Table 6:** Non-cancer minimum risk levels (MRLs) for acute and chronic toxicity in units of mg/kg/day. MRLs were not available for dermal routes of exposure.

Chemical	Oral (mg/kg/day)			Inhalation (mg/m^3^)	
	Acute MRL	Intermediate MRL	Chronic MRL	Acute MRL	Chronic MRL
Arsenic	0.005		0.0003		
Barium	0.2		0.2		
Vanadium		0.01		0.0008	0.0001

**Table 7:** Cancer risk from sediment, weathered oil, and tar using maximum and UCL values.

Chemical of Concern	Oral		Dermal		Inhalation	
	Max	UCL	Max	UCL	Max	UCL
Sediments						
Arsenic	2.3 × 10^−6^	1.6 × 10^−6^	1.4 × 10^−6^	1.1 × 10^−6^	5.1 × 10^−10^	3.8 × 10^−10^
benzo[a]pyrene	6.3 × 10^−7^	1.1 × 10^−8^	5.2 × 10^−6^	8.8 × 10^−8^	4.2 × 10^−12^	7.1 × 10^−14^
benz[a]anthracene	8.1 × 10^−8^	1.5 × 10^−9^	6.6 × 10^−7^	1.2 × 10^−8^	5.3 × 10^−13^	9.7 × 10^−15^
benzo[b]fluoranthene	6.2 × 10^−8^	1.0 × 10^−9^	5.1 × 10^−7^	8.5 × 10^−9^	4.1 × 10^−13^	6.8 × 10^−15^
dibenz[a,h]anthracene	4.6 × 10^−8^	1.1 × 10^−9^	3.8 × 10^−7^	8.6 × 10^−9^	3.0 × 10^−13^	7.0 × 10^−15^
Weathered Oil						
Arsenic	2.3 × 10^−6^	2.5 × 10^−7^	1.5 × 10^−6^	1.6 × 10^−7^	5.3 × 10^−10^	5.9 × 10^−11^
benzo[a]pyrene	3.0 × 10^−6^	3.4 × 10^−7^	2.5 × 10^−5^	2.8 × 10^−6^	2.0 × 10^−11^	2.3 × 10^−12^
benz[a]anthracene	1.4 × 10^−6^	1.7 × 10^−7^	1.2 × 10^−5^	1.4 × 10^−6^	9.5 × 10^−12^	1.1 × 10^−12^
benzo[b]fluoranthene	1.9 × 10^−7^	2.1 × 10^−8^	1.5 × 10^−6^	1.7 × 10^−7^	1.2 × 10^−12^	1.4 × 10^−13^
dibenz[a,h]anthracene	5.6 × 10^−8^	NC ^b^	4.6 × 10^−7^	NC	3.7 × 10^−13^	NC
Tar						
Arsenic	4.1 × 10^−8^	NC	1.4 × 10^−9^	NC	9.6 × 10^−12^	NC
benzo[a]pyrene	NA ^a^	NC	NA	NC	NA	NC
benz[a]anthracene	NA	NC	NA	NC	NA	NC
benzo[b]fluoranthene	2.6 × 10^−8^	NC	1.6 × 10^−10^	NC	1.7 × 10^−13^	NC
dibenz[a,h]anthracene	NA	NC	NA	NC	NA	NC

NA ^a^ = Not Available due to all samples measuring at BDL values or no measures for the matrix; NC ^b^ = Not Computed due to too few samples for determining the 95% URL.

## Supplementary Materials

The following are available online at www.mdpi.com/1660-4601/13/9/853/s1, Table S1. Non-target tentatively identified compounds in beach sands and tar collected from Grand Isle beaches. Order of chemicals in terms of highest levels to lowest.
