# Supplementary material for: Risk Assessment for Children Exposed to Beach Sands Impacted by Oil Spill Chemicals
Source: Int J Environ Res Public Health. 2016 Aug 27;13(9):853. doi: 10.3390/ijerph13090853 (PMC5036686; doi:10.3390/ijerph13090853)
Supplement: Supplementary file 1 [file ijerph-13-00853-s001.pdf]

Supplementary Materials: Risk Assessment for Children Exposed to Beach Sands Impacted by Oil Spill Chemicals

Jennifer C. Black, Jennifer N. Welday, Brian Buckley, Alesia Ferguson, Patrick L. Gurian, Kristina D. Mena, Ill Yang, Elizabeth McCandlish and Helena M. Solo-Gabriele

Table S1. Non-target tentatively identified compounds in beach sands and tar collected from Grand Isle beaches. Order of chemicals in terms of highest levels to lowest.

CAS Number	Chemical Name	Grand Isle State Park						Grand Isle Beach		
		Inter-Tidal_1	Inter-Tidal_2	Dune	Inter-Tidal_1 Targeted	Inter-Tidal_2 Targeted	Bottom of Dune	Top of Dune	Bottom of Dune Targeted	Mix of 3 Tarballs
108-88-3	Toluene	16.726	3.806	1.090	0.069	1.927	0.110	0.962	0.072	9.307
None	Oxime-, methoxy-phenyl-	0.000	0.027	2.059	0.000	3.255	1.001	0.671	4.250	3.412
584-84-9	Benzene, 2,4-diisocyanato-1-methyl-	1.277	0.952	0.745	0.013	0.727	0.582	0.469	0.472	2.141
None	1-Methyl-1-(o-methylphenyl)-1-silacyclobutane	0.056	0.029	0.000	0.026	0.073	0.015	0.017	0.003	6.516
629-62-9	Pentadecane	0.040	0.059	0.000	0.039	4.965	0.048	0.033	0.024	0.094
1190-34-7	5-Hepten-3-one, 5-methyl-	0.443	0.302	1.407	0.149	0.170	0.312	0.198	0.359	1.805
496-11-7	Indane	0.000	0.012	0.000	0.000	0.000	0.000	0.000	0.000	4.839
5400-75-9	2H-Benzimidazol-2-one, 1,3-dihydro-5-methyl-	0.482	0.440	0.354	0.380	0.475	0.366	0.329	0.336	1.477
2461-15-6	Oxirane, [[(2-ethylhexyl)oxy]methyl]-	0.169	0.251	0.327	0.297	0.023	0.110	0.180	0.008	2.919
None	4b,8-Dimethyl-2-isopropylphenanthrene, 4b,5,6,7,8,8a,9,10-octahydro-	0.000	0.000	0.000	0.000	0.000	0.000	0.000	0.000	3.953
110-54-3	n-Hexane	0.552	0.215	0.040	0.019	0.022	0.008	0.008	0.000	2.305
192823-15-7	Decane, 2,3,5,8-tetramethyl-	0.159	0.182	0.477	0.351	0.103	0.052	0.056	0.000	1.464
544-76-3	Hexadecane	0.159	0.182	0.962	0.351	0.000	0.052	0.099	0.000	0.950
106-42-3	p-Xylene	0.049	0.029	0.046	0.000	0.472	0.000	0.042	0.000	2.068
719-22-2	2,5-Cyclohexadiene-1,4-dione, 2,6-bis(1,1-dimethylethyl)-	0.680	0.435	0.106	0.013	0.020	0.840	0.295	0.026	0.094
3934-87-0	3,4-Dihydroxy-5-methoxybenzaldehyde	0.153	1.286	0.165	0.000	0.019	0.342	0.518	0.016	0.000
100-41-4	Ethylbenzene	0.022	0.000	0.000	0.000	0.114	0.000	0.000	0.000	1.984
488-23-3	Benzene, 1,2,3,4-tetramethyl-	0.000	0.095	0.939	0.351	0.000	0.052	0.099	0.000	0.453
55320-57-5	Hexanal, 3,3-dimethyl-	0.086	0.080	0.628	0.222	0.015	0.027	0.086	0.000	0.815
112-50-5	Ethanol, 2-[2-(2-ethoxyethoxy)ethoxy]-	0.040	0.007	0.000	0.000	0.015	0.000	0.005	0.000	1.846
597-49-9	3-Pentanol, 3-ethyl-	1.362	0.260	0.144	0.000	0.000	0.000	0.115	0.000	0.000
2371-19-9	2-Heptanone, 3-methyl-	0.032	0.029	0.046	0.000	0.472	0.000	0.042	0.000	1.175
96-76-4	Phenol, 2,4-bis(1,1-dimethylethyl)-	0.019	0.105	0.000	0.042	1.275	0.056	0.009	0.096	0.105
108-67-8	Mesitylene	0.008	0.020	0.965	0.000	0.092	0.033	0.010	0.000	0.554
None	2,6-Difluoro-3-methylbenzamide, *N*-(1-naphthyl)-	0.098	0.103	0.125	0.146	0.152	0.103	0.093	0.099	0.686

Table S1. *Cont.*

CAS Number	Chemical Name	Grand Isle State Park						Grand Isle Beach		
		Inter-Tidal_1	Inter-Tidal_2	Dune	Inter-Tidal_1 Targeted	Inter-Tidal_2 Targeted	Bottom of Dune	Top of Dune	Bottom of Dune Targeted	Mix of 3 Tarballs
320784-52-9	Propan-2-ol, 1-(2-isopropyl-5-methylcyclohexyloxy)-3-(4-morpholyl)-	0.026	0.050	0.077	0.125	0.187	0.077	0.090	0.150	0.584
108-38-3	Benzene, 1,3-dimethyl-	0.031	0.017	0.000	0.000	0.159	0.000	0.014	0.000	1.016
108-38-3	m-Xylene	0.029	0.017	0.000	0.000	0.159	0.000	0.014	0.000	1.014
15869-87-1	Octane, 2,2-dimethyl-	0.099	0.030	0.974	0.010	0.000	0.048	0.052	0.000	0.000
552-96-5	2,4,6-Cycloheptatrien-1-one, 2-hydroxy-5-(3-methyl-2-butenyl)-4-(1-methylethenyl)-	0.212	0.090	0.134	0.000	0.000	0.387	0.279	0.003	0.000
None	5-Methyl-2-(2-methyl-2-tetrahydrofuryl)tetrahydrofuran	0.017	0.039	0.780	0.008	0.009	0.010	0.010	0.000	0.207
None	17.alfa.,21.beta.-28,30-Bisnorhopane	0.000	0.003	0.000	0.030	0.013	0.000	0.000	0.000	1.032
74630-16-3	Phosphonous dichloride, (1,7,7-trimethylbicyclo[2.2.1]hept-2-yl)-	0.254	0.101	0.213	0.000	0.000	0.249	0.262	0.000	0.000
150544-04-0	Indole-2-one, 2,3-dihydro-6-amino-	0.126	0.126	0.098	0.110	0.053	0.089	0.079	0.031	0.304
None	3-Methylpyrazolo[bis(9-borabicyclo[3.3.1]nona-9-yl)thio]oxide	0.000	0.000	0.000	0.000	0.000	0.000	0.000	0.000	0.908
55517-88-9	Benzene, 1,4-dihexadecyl-	0.039	0.040	0.267	0.080	0.030	0.013	0.017	0.000	0.422
56554-64-4	Hexadecane, 1,1-bis(dodecyloxy)-	0.000	0.000	0.165	0.725	0.000	0.000	0.000	0.000	0.000
41898-89-9	2,3-Heptadien-5-yne, 2,4-dimethyl-	0.000	0.006	0.294	0.000	0.051	0.000	0.000	0.000	0.490
1120-21-4	Undecane	0.099	0.032	0.144	0.010	0.000	0.048	0.052	0.011	0.391
18479-57-7	2-Octanol, 2,6-dimethyl-	0.023	0.033	0.045	0.000	0.000	0.034	0.601	0.032	0.000
87020-51-7	Octadecanoic acid, tert-butyldimethylsilyl ester	0.074	0.159	0.000	0.025	0.000	0.109	0.000	0.164	0.222
14411-56-4	Benzene, 1-(1,1-dimethylethyl)-3-ethyl-	0.085	0.127	0.454	0.000	0.000	0.000	0.050	0.000	0.000
16747-25-4	Hexane, 2,2,3-trimethyl-	0.016	0.000	0.015	0.018	0.008	0.000	0.086	0.000	0.570
74685-33-9	3-Eicosene, (E)-	0.000	0.016	0.000	0.000	0.621	0.000	0.045	0.006	0.000
98-88-4	Benzoyl chloride	0.017	0.000	0.267	0.000	0.130	0.000	0.000	0.000	0.254
78706-77-1	1-Butanone, 2-chloro-3-methyl-1-phenyl-	0.017	0.041	0.005	0.000	0.130	0.000	0.000	0.000	0.471
527-53-7	Benzene, 1,2,3,5-tetramethyl-	0.000	0.005	0.006	0.000	0.013	0.000	0.000	0.000	0.633
None	Benzamide, *N*-(3-methylthio-1,2,4-thiadiazol-5-yl)-	0.000	0.041	0.000	0.000	0.082	0.000	0.000	0.052	0.471
99-87-6	p-Cymene	0.007	0.000	0.018	0.000	0.008	0.000	0.000	0.000	0.570
59212-75-8	2,2′-Isopropylidenebis(5-methylfuran)	0.000	0.000	0.094	0.134	0.233	0.000	0.000	0.140	0.000
92-52-4	Biphenyl	0.009	0.007	0.007	0.010	0.010	0.014	0.003	0.004	0.535
585-74-0	Ethanone, 1-(3-methylphenyl)-	0.000	0.000	0.000	0.000	0.000	0.000	0.000	0.000	0.585
693-54-9	2-Decanone	0.018	0.066	0.081	0.000	0.177	0.000	0.044	0.000	0.196
40710-70-1	Octatetracontane, 1-iodo-	0.000	0.015	0.433	0.015	0.045	0.000	0.000	0.010	0.054
52896-87-4	Heptane, 4-(1-methylethyl)-	0.018	0.013	0.015	0.000	0.000	0.052	0.000	0.000	0.453
1565-81-7	3-Decanol	0.041	0.000	0.362	0.000	0.000	0.021	0.000	0.000	0.099
112-39-0	Hexadecanoic acid, methyl ester	0.040	0.033	0.041	0.060	0.056	0.005	0.033	0.042	0.194

Table S1. *Cont.*

CAS Number	Chemical Name	Grand Isle State Park						Grand Isle Beach		
		Inter-Tidal_1	Inter-Tidal_2	Dune	Inter-Tidal_1 Targeted	Inter-Tidal_2 Targeted	Bottom of Dune	Top of Dune	Bottom of Dune Targeted	Mix of 3 Tarballs
None	1-Adamantanecarboxylic acid, 2-tetrahydrofurylmethyl ester	0.058	0.013	0.167	0.013	0.041	0.002	0.008	0.015	0.166
91-20-3	Naphthalene	0.145	0.055	0.039	0.034	0.070	0.016	0.112	0.011	0.000
30221-21-7	tert-Butyl isopropyl carbonate	0.112	0.000	0.122	0.100	0.015	0.000	0.031	0.000	0.099
445-27-2	o-Fluoroacetophenone	0.000	0.000	0.000	0.072	0.368	0.006	0.000	0.030	0.000
137235-48-4	6S-2,3,8,8-Tetramethyltricyclo[5.2.2.0(1,6)]undec-2-ene	0.006	0.003	0.095	0.133	0.233	0.000	0.000	0.000	0.000
4426-31-7	Borinic acid, diethyl-	0.039	0.007	0.286	0.000	0.130	0.000	0.000	0.000	0.000
33933-82-3	2-Decanone, 5,9-dimethyl-	0.000	0.000	0.000	0.204	0.015	0.067	0.055	0.112	0.000
None	1H-Pyrazolo[3,4-b]pyridin-3(2H)-one, 6-hydroxy-4-methyl-2-phenyl-	0.000	0.012	0.000	0.000	0.000	0.000	0.000	0.000	0.392
17699-05-7	Bicyclo[3.1.1]hept-2-ene, 2, 6-dimethyl-6-(4-methyl-3- pentenyl)-	0.000	0.000	0.115	0.042	0.143	0.000	0.000	0.087	0.000
52-53-9	Verapamil	0.000	0.000	0.000	0.014	0.032	0.000	0.000	0.000	0.337
121747-63-5	2,2,6,7-Tetramethyl-10-oxatricyclo[4.3.0.1(1,7)]decan-one	0.058	0.034	0.060	0.014	0.029	0.043	0.072	0.054	0.000
34645-03-9	3,6-Dimethyl-4-octanone	0.000	0.000	0.000	0.000	0.056	0.000	0.014	0.000	0.294
111-13-7	2-Octanone	0.000	0.154	0.000	0.000	0.182	0.000	0.023	0.000	0.000
620-14-4	Benzene, 1-ethyl-3-methyl-	0.000	0.000	0.000	0.000	0.018	0.000	0.000	0.000	0.338
None	8H-Pyrano[2,3-e]benzothiophen-8-one, 4-formamido-6-methyl	0.000	0.000	0.000	0.000	0.000	0.000	0.000	0.000	0.349
84609-99-4	Pyrimidin-5-amine, 2-(4-butoxyphenyl)-	0.000	0.000	0.000	0.000	0.000	0.000	0.000	0.000	0.349
3910-35-8	1H-Indene, 2,3-dihydro-1,1,3-trimethyl-3-phenyl-	0.058	0.026	0.051	0.024	0.061	0.068	0.053	0.009	0.000
2371-42-8	2-Methylisoborneol	0.015	0.025	0.228	0.000	0.066	0.000	0.014	0.000	0.000
98-82-8	Isopropylbenzene	0.000	0.040	0.000	0.000	0.036	0.000	0.000	0.000	0.272
155249-75-5	3-Chloro-*N*,*N*-dimethyl-6-phenyl-11H-indolo[3,2-c] quinoline-11-ethanamine, 5-oxide	0.007	0.007	0.061	0.040	0.026	0.012	0.012	0.021	0.145
85-01-8	Phenanthrene	0.030	0.018	0.013	0.014	0.057	0.007	0.005	0.009	0.156
122-03-2	Benzaldehyde, 4-(1-methylethyl)-	0.019	0.007	0.000	0.000	0.009	0.004	0.000	0.003	0.260
10152-65-5	Cyclopropanedodecanoic acid, 2-octyl-, methyl ester	0.003	0.007	0.000	0.000	0.007	0.004	0.000	0.003	0.260
700-12-9	Benzene, pentamethyl-	0.000	0.000	0.000	0.000	0.105	0.000	0.000	0.000	0.178
63922-41-8	5-Hepten-3-yn-2-ol, 6-methyl-5-(1-methylethyl)-	0.075	0.045	0.014	0.000	0.000	0.120	0.027	0.000	0.000
3/7/3983	Cyclohexene,1-(2-methylpropyl)-	0.000	0.000	0.000	0.000	0.000	0.000	0.000	0.000	0.281
2471-83-2	1H-Indene, 1-ethylidene-	0.000	0.000	0.000	0.007	0.014	0.000	0.000	0.000	0.230
None	Sulfurous acid, octyl 2-pentyl ester	0.094	0.005	0.120	0.000	0.000	0.011	0.013	0.007	0.000
29812-79-1	Hydroxylamine, O-decyl-	0.012	0.014	0.000	0.000	0.201	0.000	0.023	0.000	0.000
84534-30-5	3-Heptadecanol	0.009	0.029	0.030	0.000	0.000	0.004	0.176	0.000	0.000
82756-19-2	Benzo[b][1,8]-naphthyridin-5(10H)-one, 2,4,7-trimethyl-	0.000	0.000	0.000	0.000	0.000	0.000	0.000	0.000	0.240
29211-68-5	3-tert-Butyl-2-pyrazolin-5-one	0.021	0.044	0.029	0.000	0.022	0.031	0.014	0.079	0.000

Table S1. *Cont.*

CAS Number	Chemical Name	Grand Isle State Park						Grand Isle Beach		
		Inter-Tidal_1	Inter-Tidal_2	Dune	Inter-Tidal_1 Targeted	Inter-Tidal_2 Targeted	Bottom of Dune	Top of Dune	Bottom of Dune Targeted	Mix of 3 Tarballs
91-57-6	2-Methylnaphthalene	0.000	0.000	0.000	0.000	0.021	0.000	0.004	0.000	0.213
112899-61-3	2-Naphthalenol,6,7-epoxy-5,6,7,8-tetrahydro-5-isopropyl-3-methyl-8-methylene-	0.044	0.018	0.029	0.000	0.000	0.081	0.063	0.000	0.000
3777-69-3	Furan, 2-pentyl-	0.000	0.000	0.000	0.028	0.155	0.000	0.000	0.052	0.000
54699-29-5	Cyclohexane, (ethoxymethoxy)-	0.024	0.029	0.030	0.007	0.000	0.009	0.133	0.000	0.000
504-20-1	Phorone	0.032	0.007	0.000	0.034	0.133	0.011	0.008	0.003	0.000
7580-85-0	Ethanol, 2-(1,1-dimethylethoxy)-	0.012	0.029	0.000	0.028	0.137	0.000	0.023	0.000	0.000
60761-23-1	Tetrahydroionone	0.000	0.000	0.000	0.000	0.097	0.000	0.000	0.000	0.121
95-47-6	o-Xylene	0.053	0.000	0.063	0.000	0.000	0.000	0.096	0.000	0.000
98-82-8	Isopropylbenzene	0.000	0.000	0.000	0.000	0.153	0.000	0.000	0.052	0.000
54965-53-6	1-Propanone, 2-chloro-1-(2,4-dimethylphenyl)-2-methyl-	0.015	0.015	0.000	0.017	0.000	0.010	0.000	0.007	0.135
115754-79-5	3-(1-Cyclopentenyl)furan	0.000	0.000	0.000	0.000	0.000	0.000	0.000	0.000	0.183
2408-37-9	Cyclohexanone, 2,2,6-trimethyl-	0.000	0.006	0.000	0.000	0.177	0.000	0.000	0.000	0.000
475-03-6	Naphthalene, 1,2,3,4-tetrahydro-1,1,6-trimethyl-	0.000	0.000	0.000	0.000	0.006	0.000	0.000	0.000	0.173
57681-51-3	2(3H)-Furanone, 5-(acetyloxy)dihydro-5-methyl-	0.000	0.014	0.000	0.000	0.133	0.000	0.023	0.000	0.000
624-42-0	3-Heptanone, 6-methyl-	0.000	0.014	0.000	0.000	0.133	0.000	0.014	0.000	0.000
102281-26-5	α-L-Fucopyranose 1,2:3,4-bis(benzeneboronate)	0.000	0.000	0.000	0.088	0.000	0.000	0.000	0.000	0.059
None	3-Ethyl-2-pentadecanone	0.000	0.000	0.086	0.000	0.000	0.000	0.045	0.015	0.000
33156-93-3	6-[(Z)-1-Butenyl]-1,4-cycloheptadiene	0.006	0.000	0.000	0.000	0.118	0.000	0.017	0.000	0.000
89-74-7	Ethanone, 1-(2,4-dimethylphenyl)-	0.015	0.004	0.000	0.000	0.007	0.000	0.000	0.000	0.113
10203-30-2	3-Dodecanol	0.018	0.009	0.000	0.000	0.000	0.048	0.060	0.000	0.000
16466-24-3	2(3H)-Furanone, dihydro-4,4,5,5-tetramethyl-	0.041	0.007	0.009	0.000	0.000	0.018	0.047	0.012	0.000
5129-58-8	Tridecanoic acid, 12-methyl-, methyl ester	0.011	0.008	0.011	0.014	0.012	0.008	0.009	0.011	0.043
85763-57-1	11-Methyldodecanol	0.000	0.000	0.000	0.007	0.091	0.000	0.000	0.000	0.000
20548-62-3	Phthalic acid, bis(7-methyloctyl) ester	0.000	0.006	0.015	0.014	0.011	0.007	0.005	0.000	0.035
120-12-7	Anthracene	0.030	0.018	0.000	0.000	0.035	0.000	0.000	0.009	0.000
541-73-1	1,3-Dichlorobenzene	0.026	0.006	0.028	0.000	0.000	0.011	0.021	0.000	0.000
65925-28-2	Benzene, 1-[2-(2-chloroethoxy)ethoxy]-4-(1,1,3,3-tetramethylbutyl)-	0.000	0.000	0.000	0.000	0.000	0.000	0.000	0.000	0.091
627-73-6	Butanedioic acid, ethyl methyl ester	0.011	0.000	0.000	0.000	0.032	0.015	0.032	0.000	0.000
70987-81-4	1-(1,2,3-Trimethyl-cyclopent-2-enyl)-ethanone	0.000	0.071	0.000	0.000	0.000	0.000	0.000	0.014	0.000
829-26-5	Naphthalene, 2,3,6-trimethyl-	0.000	0.000	0.004	0.000	0.000	0.000	0.002	0.000	0.075
1119-44-4	3-Hepten-2-one	0.000	0.075	0.000	0.000	0.000	0.003	0.000	0.000	0.000
100807-88-3	Butyric acid, 6-(4-cyano-phenyl)-naphthalen-2-yl ester	0.000	0.000	0.000	0.000	0.000	0.000	0.000	0.000	0.073

Table S1. *Cont.*

CAS Number	Chemical Name	Grand Isle State Park						Grand Isle Beach		
		Inter-Tidal_1	Inter-Tidal_2	Dune	Inter-Tidal_1 Targeted	Inter-Tidal_2 Targeted	Bottom of Dune	Top of Dune	Bottom of Dune Targeted	Mix of 3 Tarballs
122-03-2	Benzaldehyde, 4-(1-methylethyl)-	0.015	0.007	0.000	0.000	0.007	0.000	0.000	0.000	0.041
20185-55-1	Benzoic acid, 4-(1-methylethyl)-, methyl ester	0.000	0.000	0.015	0.000	0.000	0.000	0.000	0.000	0.049
61141-65-9	Benzocyclododecene, 2,3-diethyl-4a,5,6,7,8,9,10,11,12,13-decahydro-	0.010	0.000	0.007	0.000	0.000	0.015	0.013	0.014	0.000
2700-84-7	2,2-Dimethyl-1-(2,4,6-trimethylphenyl)propan-1-one	0.000	0.000	0.000	0.000	0.022	0.000	0.000	0.033	0.000
13956-29-1	Cannabidiol	0.004	0.000	0.005	0.000	0.000	0.024	0.022	0.000	0.000
18835-33-1	1-Hexacosene	0.000	0.000	0.000	0.000	0.019	0.031	0.000	0.000	0.000
None	15-Hydroxy-7-oxodehydroabietic acid, methyl ester	0.000	0.000	0.000	0.000	0.000	0.000	0.000	0.000	0.049
127-43-5	1-Penten-3-one, 1-(2,6,6-trimethyl-1-cyclohexen-1-yl)-	0.000	0.000	0.000	0.000	0.000	0.000	0.000	0.000	0.046
106-46-7	Benzene, 1,4-dichloro-	0.019	0.004	0.000	0.000	0.000	0.008	0.013	0.000	0.000
829-26-5	Naphthalene, 2,3,6-trimethyl-	0.000	0.000	0.000	0.000	0.000	0.000	0.000	0.000	0.043
29577-19-3	m-Cymene, 5-tert-butyl-	0.000	0.000	0.000	0.000	0.000	0.000	0.000	0.000	0.036
6909-22-4	5-Caranol, trans,trans-(+)-	0.000	0.000	0.000	0.000	0.029	0.000	0.000	0.000	0.000
None	Androst-5-en-3-ol-17-one, 16,16-trimethylenedithio-	0.005	0.000	0.000	0.000	0.000	0.005	0.018	0.000	0.000
None	1-Benzenesulfonohydrazide, *N*′-tricyclo[4.2.2.0(1,5)dec-8-ylidene]	0.000	0.000	0.000	0.007	0.000	0.000	0.000	0.000	0.000
925-78-0	3-Nonanone	0.000	0.005	0.000	0.000	0.000	0.000	0.000	0.000	0.000
21898-95-3	Tricyclo[4.3.1.1(3,8)]undecane, 1-methoxy-	0.005	0.000	0.000	0.000	0.000	0.000	0.000	0.000	0.000
29812-79-1	Hydroxylamine, O-decyl-	0.000	0.000	0.000	0.000	0.000	0.000	0.000	0.000	0.000
544-76-3	Hexadecane	0.000	0.000	0.000	0.000	0.000	0.000	0.000	0.000	0.000
None	9-Methylene-1-phenyl-3,6-diazahomoadaman	0.000	0.000	0.000	0.000	0.000	0.000	0.000	0.000	0.000
	**Sum (ng/g)**	**25.1**	**10.9**	**17.3**	**4.8**	**20.0**	**6.0**	**7.4**	**7.1**	**68.6**

© 2016 by the authors; licensee MDPI, Basel, Switzerland. This article is an open access article distributed under the terms and conditions of the Creative Commons by Attribution (CC-BY) license (http://creativecommons.org/licenses/by/4.0/).
